# Promoter Methylation–Expression Coupling of Gliogenesis Genes in *IDH*-Wildtype Glioblastoma: Longitudinal Analysis and Prognostic Value

**DOI:** 10.3390/ijms27021112

**Published:** 2026-01-22

**Authors:** Roxana Radu, Ligia Gabriela Tataranu, Anica Dricu, Oana Alexandru

**Affiliations:** 1 Department of Neurosurgery, Faculty of Medicine, Carol Davila University of Medicine and Pharmacy, 020021 Bucharest, Romania; roxana.radu@outlook.com (R.R.); ligia.tataranu@umfcd.ro (L.G.T.); 2 Department of Neurosurgery, Bagdasar-Arseni Clinical Emergency Hospital, 041915 Bucharest, Romania; 3 Department of Biochemistry, Faculty of Medicine, Carol Davila University of Medicine and Pharmacy, 020021 Bucharest, Romania; 4 Department of Neurology, Faculty of Medicine, University of Medicine and Pharmacy Craiova, 200349 Craiova, Romania; oanale@hotmail.com

**Keywords:** glioblastoma, *IDH*-wildtype, DNA methylation, gliogenesis, promoter regulation, prognosis

## Abstract

Glioblastoma (GBM) shows extensive epigenetic heterogeneity. In *IDH*-wildtype (*IDH*-WT) GBM, promoter DNA methylation may regulate lineage programs influencing tumor evolution and prognosis; here, we systematically profiled promoter-level methylation dynamics across longitudinal tumors. Genome-wide DNA methylation data were obtained from the publicly available Gene Expression Omnibus (GEO; GSE279073) dataset, comprising a longitudinal cohort of 226 *IDH*-wildtype glioblastomas profiled on the Illumina Infinium EPIC 850K array across primary and recurrent stages at the University of California, San Francisco. From 333 Gene Ontology gliogenesis-annotated genes (GO:0042063), a 48-gene promoter panel was derived, with ≥2 probes per gene. Promoter methylation was summarized as the median β-value and tested using one-sample Wilcoxon with FDR correction. Functional enrichment, longitudinal variation, and patient-level methylation burden were assessed. Validation analyses were performed using independent *IDH*-wildtype GBM datasets from The Cancer Genome Atlas (RNA-seq and 450K methylation; *n* = 347). Promoter hypomethylation predominated across all stages, with 25 genes consistently hypomethylated and 7 hypermethylated. Functional enrichment highlighted gliogenesis, glial cell differentiation, neurogenesis, and Notch-related signaling. In TCGA, promoter methylation inversely correlated with expression for 11 of 33 genes (FDR < 0.05). An Expression Score contrasting hypomethylated and hypermethylated genes was positively associated with improved overall survival, where higher scores predicted better outcome (HR = 0.87, *p* = 0.016; Q4 vs. Q1 HR = 0.68, *p* = 0.025), and a complementary Methylation Score showed that higher promoter hypermethylation predicted poorer outcome (HR = 1.73, *p* < 0.001). *CNTN2* and *TSPAN2* were adverse prognostic genes (FDR < 0.05). The Expression Score was highest in Proneural tumors and lowest in Mesenchymal tumors (*p* < 0.001), reflecting a proneural-like state associated with better prognosis. Promoter methylation within gliogenesis genes defines a stable yet prognostically informative epigenetic signature in *IDH*-WT GBM. Hypomethylation promotes transcriptional activation and a favorable outcome, whereas hypermethylation represses lineage programs and predicts poorer survival.

## 1. Introduction

Glioblastoma (GBM) remains the most common and lethal primary malignant brain tumor in adults. In the 2021 fifth edition of the World Health Organization (WHO) classification (CNS5), diffuse gliomas were reorganized with molecular criteria taking center stage; under this schema, glioblastoma is restricted to *IDH*-wildtype (*IDH*-WT) tumors and can be diagnosed by histologic features or, in the appropriate setting, by the presence of a telomerase reverse-transcriptase promoter (*TERT*-p) mutation, *EGFR* amplification, or the combined whole-chromosome copy-number change of +7/−10, even in the absence of classic histology [1,2,3]. Beyond this classification, Capper et al. demonstrated that DNA methylation profiling can refine diagnoses and reassign a meaningful fraction of cases. The approach has since been widely adopted in specialized centers [4]. At the gene level, promoter CpG methylation is a canonical repressive mark that can prevent transcription-factor binding and recruit repressive chromatin, though context-specific exceptions exist [5,6,7,8].

Glial fate specification is relevant to GBM, with Notch signaling and allied pathways orchestrating the transition from neurogenesis to gliogenesis, maintaining neural stem/progenitor pools, and regulating cell differentiation. Accordingly, gliogenesis genes provide a developmental framework frequently co-opted in gliomagenesis [9,10,11].

Longitudinally, methylation classes are often stable at recurrence, yet focal methylation changes and treatment-linked remodeling (e.g., under temozolomide) are reported, with substantial patient-level heterogeneity. The extent to which promoter changes in gliogenesis genes show coherent transcriptional effects and relate to outcome in *IDH*-WT GBM remains incompletely defined [12,13,14,15]. We address this gap by focusing on promoter DNA methylation–expression coherence within a curated set of gliogenesis genes in *IDH*-WT GBM. Using a longitudinal cohort spanning primary and recurrent tumors, we quantify stage-specific and cross-stage patterns of promoter hypo- and hypermethylation, exploit within-patient paired comparisons to probe change across recurrences, characterize patient-level heterogeneity, and assess transcriptional coherence and prognostic associations in external data. We aim to determine whether gliogenesis promoters carry a stable epigenetic “footprint” across recurrences and whether methylation–expression coupling within this program encodes clinically relevant risk in *IDH*-WT GBM.

## 2. Results

### 2.1. Coverage of the Gliogenesis Universe and Statistical Implications

From the 333 GO-annotated gliogenesis genes (GO:0042063), 328 (98.6%) were represented by at least one EPIC probe in GSE279073, indicating near-complete platform coverage of the biological universe under study. We defined the promoter a priori as probes annotated in the Illumina manifest to TSS1500, TSS200, 5′UTR, or 1stExon. Requiring ≥ 2 promoter probes per gene and summarizing promoter methylation as the median β value yielded a robust, inference-ready 48-gene promoter panel—14.4% of the 333-gene universe (or 14.6% of the 328 array-represented genes). An all-stage heatmap of these 48 genes visualizes the pattern across primary, first recurrence (Recur1), and second recurrence (Recur2) and demonstrates the broad hypomethylation signature that persists through recurrence (Figure S1).

### 2.2. Stage-Wise Prevalence of Promoter Hypomethylation and Hypermethylation

**Figure 1:**
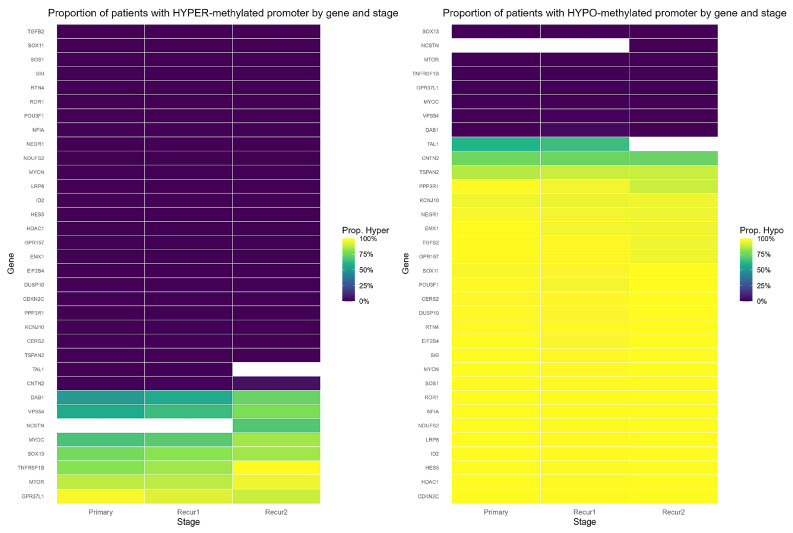
Stage-wise prevalence of promoter methylation. Heatmaps show the proportion of tumors with hypermethylation (**left**) or hypomethylation (**right**) per gene across Primary, Recurrent1, and Recurrent2 (purple → yellow = 0 → 1). Gene-level calls were defined vs. β = 0.50 by one-sample Wilcoxon (BH) with |Δβ| ≥ 0.20; only significant genes are displayed. Hypomethylation predominates across stages, whereas hypermethylation is less frequent.

Across primary, first, and second tumor recurrence, promoter hypomethylation predominated. Specifically, 26/48 (54.2%) were hypomethylated and 7/48 (14.6%) hypermethylated in primary; 26/48 hypomethylated and 7/48 hypermethylated in first recurrence; and 25/48 (52.1%) hypomethylated with 8/48 (16.7%) hypermethylated in second recurrence (Table S1). Heatmaps of the proportion of patients carrying hypo-/hyper-methylation calls per gene corroborate the stability of these patterns across stages (Figure 1). As pre-specified, the third recurrence (*n* = 2) was excluded from inference and used descriptively.

For each gene and stage, we tested whether the promoter (median β value across promoter probes) differs from 0.50 using a one-sample Wilcoxon signed-rank test with Benjamini–Hochberg FDR; calls required |Δβ| = |β_median_ − 0.50| ≥ 0.20. At primary, most significant promoters showed large negative Δβ (Wilcoxon *p* < 0.001; FDR < 0.001). The pattern persisted at first recurrence (*p* < 0.001; FDR < 0.001). At the second recurrence, fewer genes met thresholds, but the direction was consistent (e.g., *TSPAN2* hypomethylated; *TNFRSF1B* and *VPS54* hypermethylated; all *p* < 0.001; FDR < 0.001). Per-gene β, Δβ, Wilcoxon p, and FDR are summarized in Table S2A–C.

Intersections across primary, first, and second tumor recurrence revealed a large core of consistent promoters: 25/48 hypomethylated and 7/48 hypermethylated were called in all three stages. A full presence matrix (gene × stage × call) is provided in Table S3.

Consistent hypomethylated promoters (25 genes): *CDKN2C*, *CERS2*, *CNTN2*, *DUSP10*, *EIF2B4*, *EMX1*, *GPR157*, *HDAC1*, *HES5*, *ID2*, *KCNJ10*, *LRP8*, *MYCN*, *NDUFS2*, *NEGR1*, *NFIA*, *POU3F1*, *PPP3R1*, *ROR1*, *RTN4*, *SKI*, *SOS1*, *SOX11*, *TGFB2*, *TSPAN2*.

Consistent hypermethylated promoters (7 genes): *DAB1*, *GPR37L1*, *MTOR*, *MYOC*, *SOX13*, *TNFRSF1B*, *VPS54*.

Gene-level methylation calls derived from individual promoter subregions showed high concordance with the combined promoter definition (mean concordance 0.78–0.82 across bins), indicating that promoter subregion heterogeneity does not dilute the observed methylation signal (Table S4).

### 2.3. Burden Heterogeneity and Within-Patient Methylation Changes

**Figure 2:**
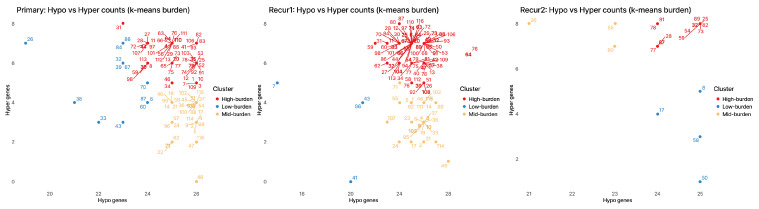
Tumor-level burden of promoter methylation across stages. Scatterplots for Primary, Recur1, and Recur2. Each dot is one tumor; *x*-axis = number of hypomethylated promoters, *y*-axis = number of hypermethylated promoters (of 48). Patients are clustered into three burden groups—low, mid, and high—colored blue (low), orange (mid), and red (high). Arrows connect longitudinal samples from the same patient across stages. The plots visually separate the three burden groups and suggest a higher overall burden at later recurrence. Sample size varies by stage (fewer tumors at second recurrence), so the Recur2 panel contains fewer points.

We counted hypomethylation and hypermethylation promoter calls per patient and clustered patients into three groups (low, mid, and high burden). The distributions differed significantly between clusters at every stage (Kruskal–Wallis test; for hypomethylation: primary and first recurrence *p* < 0.001, second recurrence *p* = 0.011; for hypermethylation: primary and first recurrence *p* < 0.001, second recurrence *p* = 0.0039). Across stages, the overall burden increased with recurrence (Kruskal–Wallis test across stages: hypomethylation *p* = 0.0037; hypermethylation *p* < 0.001). Pairwise comparisons confirmed higher burden at the second recurrence, with hypomethylation greater at second recurrence than at primary (*p* = 0.0033) and then at first recurrence (*p* = 0.0018), and hypermethylation greater at second recurrence than at both primary and first recurrence (both *p* < 0.001). The scatterplots illustrate the separation of the three burden groups (Figure 2), and statistical details are shown in Table S5A–D. Silhouette analysis supported the use of three burden groups, with k = 3 yielding the highest average silhouette width in primary and second recurrence tumors and comparable performance in first recurrence (Table S6).

**Figure 3:**
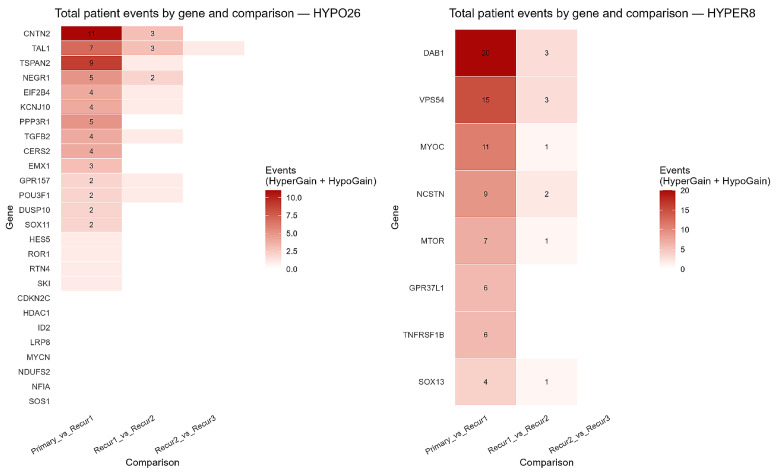
Longitudinal promoter-methylation events (gains). Heatmaps show, by gene (rows) and adjacent-stage comparison (Primary → Recur1, Recur1 → Recur2, Recur2 → Recur3), the number of patients who newly acquired a promoter call (HypoGain or HyperGain; color = count). (**Left**): HYPO set (*n* = 26). (**Right**): HYPER set (*n* = 8). Only genes with ≥1 event are shown; Recur2 → Recur3 counts reflect the smaller sample size.

For each available patient, we counted HyperGain events (Δβ ≥ +0.20) and HypoGain events (Δβ ≤ −0.20) between consecutive stages and summed them per gene. The most significant number of events occurred from primary to first recurrence, fewer from first to second recurrence, and only isolated single events from second to third recurrence (there were only two third-recurrence transitions). Within the hypermethylated subset, the leading contributors were *DAB1* (20 events on primary → first recurrence; 3 on first → second), *VPS54* (15; 3), *MYOC* (11; 1), *NCSTN* (9; 2), and *MTOR* (7; 1). Within the hypomethylated subset, the leading contributors were *CNTN2* (11; 3), *TAL1* (7; 3; and one isolated event on second → third), *TSPAN2* (9; 1), and *NEGR1* (5; 2). These totals are shown in a compact two-panel heatmap (Figure 3). Full matrices and summaries are provided in Table S7A–G.

Formal binomial tests comparing counts of HyperGain versus HypoGain for each gene did not identify any imbalance after control of the false discovery rate (Table S8A,B).

To exemplify individual-level behavior, *NCSTN* (hypermethylated only at second recurrence) and *TAL1* (absent at second recurrence) show rare, patient-specific step changes concentrated on primary → first recurrence (Figure S2). Cohort trajectories for *NCSTN* show a positive mixed-effects trend on the M-scale (slope = 0.190; *p* = 3.75 × 10^−4^; q = 1.80 × 10^−2^; Figure S3).

Analyses on the M-value scale using linear models with duplicate-sample correlation (blocking by patient) did not detect differentially methylated promoters at a false discovery rate < 0.05 for either first recurrence versus primary or second recurrence versus first recurrence (Table S9A,B). Applying the TREAT (Testing Relative to a Threshold) procedure [16] to enforce a minimum effect size (log-fold-change threshold corresponding approximately to |Δβ| ≥ 0.20) likewise showed no significant promoters (Table S10A,B).

Agreement between the stage-wise β-based calling (one-sample Wilcoxon test with false discovery rate control and |Δβ| ≥ 0.20) and the limma decisions was perfect at every stage (Cohen’s kappa = 1.00) (Table S11A–E).

Leave-one-patient-out re-calling demonstrated high stability of significant stage calls. The median retention proportion was 1.00 at primary, first recurrence, and second recurrence, and all significant calls were retained in at least 95% of resamples, except for *DAB1* at primary (hypermethylated), which retained 51% (Table S12A–D).

### 2.4. Functional Enrichment of Significant Promoters

**Figure 4:**
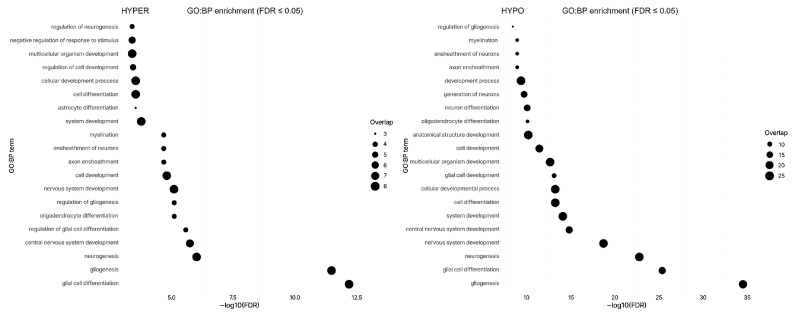
GO Biological Process enrichment for union gene sets. Horizontal dot plots show the top enriched GO-BP terms for promoters with hypermethylation (**left**) or hypomethylation (**right**). The *x*-axis gives the number of genes from the 48-gene panel annotated to each term (top hits; FDR < 0.05). HYPO genes concentrate in neuro/glial development (e.g., gliogenesis, glial cell differentiation, neurogenesis, nervous-system development), whereas HYPER genes show weaker, more general developmental/regulatory signals.

Over-representation analyses using g:Profiler with a genome-wide background showed strong enrichment for glial lineage and neural development programs across the promoter sets. In the union of all stage-significant promoters, Gene Ontology Biological Process terms were led by gliogenesis, glial cell differentiation, and neurogenesis (adjusted *p* < 0.001 throughout). Direction-stratified analyses preserved this biology: the hypomethylated-only subset was enriched for gliogenesis, glial cell differentiation, oligodendrocyte differentiation, myelination, and ensheathment of neurons (adjusted *p* < 0.001), and the hypermethylated-only subset again featured glial cell differentiation and gliogenesis among the top terms (adjusted *p* < 0.001). Complementary pathway collections (Reactome and WikiPathways) demonstrated Notch-related signaling in the union set and also in the hypermethylated-only subset (WikiPathways Notch signaling, adjusted *p* = 0.028); Notch pathway was not significant in the hypomethylated-only subset at the false-discovery-rate threshold used. These results are shown in Figure 4 and Figure S4, Tables S13 and S14A–C.

### 2.5. TCGA Validation (IDH-Wildtype Glioblastoma)

**Figure 5:**
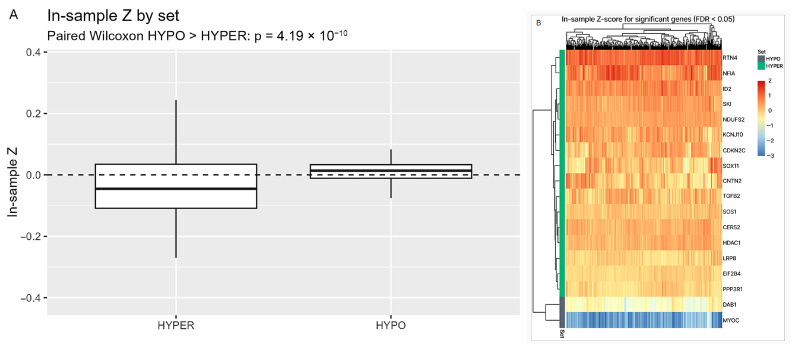
(**A**) In-sample expression Z by gene set (TCGA, *IDH*-WT). Boxplots compare per-gene in-sample Z-scores for the HYPER and HYPO sets (dashed line = 0). The HYPO set shows a positive shift relative to the HYPER set, consistent with higher expression at hypomethylated promoters. Paired Wilcoxon (HYPO > HYPER): *p* < 0.001. (**B**) Expression heatmap for significant genes (TCGA, *IDH*-WT). Rows are genes significant at FDR < 0.05; columns are tumors. Cells show in-sample expression Z-scores (blue = low, red = high). The sidebar marks the gene set (HYPO teal, HYPER gray). Genes and samples are ordered by hierarchical clustering. HYPO genes display predominantly higher expression, whereas HYPER genes show lower expression, consistent with a repressive promoter mark. The HYPER set is limited to two genes (*DAB1* and *MYOC*); therefore, this figure should be interpreted only as a directional, cross-cohort validation of promoter-associated expression patterns at the gene-set level.

After filtering the TCGA-GBM dataset to *IDH*-wildtype tumors, we analyzed *n* = 347 patients with valid overall survival data. Genes were classified as hypomethylated (HYPO) or hypermethylated (HYPER) based on promoter methylation status in GSE279073, and their expression patterns were examined in an independent TCGA cohort as a biological validation step. For each sample, we computed an expression signature score by standardizing expression within the sample (in-sample Z) and taking the difference between the hypomethylated and hypermethylated promoter sets (HYPO − HYPER). At the cohort level, hypomethylated genes were globally more highly expressed than hypermethylated genes (paired Wilcoxon test, *p* < 0.001; pseudo-median ≈ +0.049 Z), consistent with the regulatory interpretation of the promoter calls (Figure 5A). At the gene level, one-sample Wilcoxon tests against zero on the Z scale, with Benjamini–Hochberg false-discovery-rate control, identified 18 of 34 genes as significant at FDR < 0.05 and in the expected direction (HYPER lower; HYPO higher). The heatmap of significant genes and the complete statistics are shown in Figure 5B and Table S15A,B, respectively.

**Figure 6:**
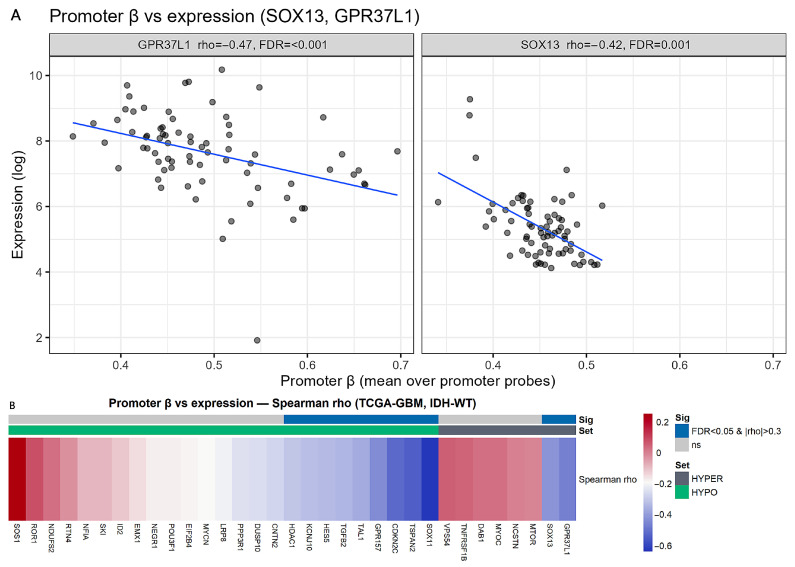
(**A**,**B**): Promoter β vs. expression coupling (TCGA, *IDH*-WT). (**A**) Scatterplots for *GPR37L1* and *SOX13* show inverse associations between promoter β (mean across promoter probes) and log expression across tumors (blue line = linear fit). Reported are Spearman ρ and FDR, demonstrating significant negative coupling (ρ < 0). (**B**) Heatmap of Spearman ρ between promoter β (median across TSS1500/TSS200/5′UTR/1stExon probes) and RNA expression for all covered genes (columns). Top bars indicate statistical significance (FDR < 0.05); among significant columns, we additionally flag moderate/strong effects with |ρ| ≥ 0.30. |ρ| ≥ 0.30 flags correlations that are at least moderate in size, regardless of sign. The sidebar indicates gene set (HYPO = teal, HYPER = gray). Most significant associations are negative (inverse coupling), consistent with a repressive promoter mark (higher β → lower expression).

Coupling between promoter methylation and expression was assessed in the subset with data from both platforms available. From the GDC 450 k array, we could match 74 tumors (barcode-15) to RNA-seq. Promoter methylation was summarized as the median β value across TSS1500, TSS200, 5′UTR, and 1stExon probes. Because the 450k array does not cover all promoters in the 48-gene panel (for example, *CERS2* lacked measurable promoter probes in this download), analyses were restricted to genes with promoter coverage. As β encodes promoter methylation (repressive), the a priori expectation was an inverse association with expression (ρ < 0) irrespective of HYPO/HYPER labels. Consistently, two hypermethylated promoters showed significant inverse coupling—*SOX13* (ρ ≈ −0.42, FDR = 0.001) and *GPR37L1* (ρ ≈ −0.47, FDR < 0.001). In addition, nine hypomethylated promoters displayed significant inverse β-expression coupling (ρ < 0, FDR < 0.05): *SOX11*, *TSPAN2*, *CDKN2C*, *GPR157*, *TAL1*, *TGFB2*, *HES5*, *KCNJ10*, and *HDAC1*—indicating higher expression at hypomethylation, as expected for a repressive promoter mark (Figure 6A,B; Table S16).

**Figure 7:**
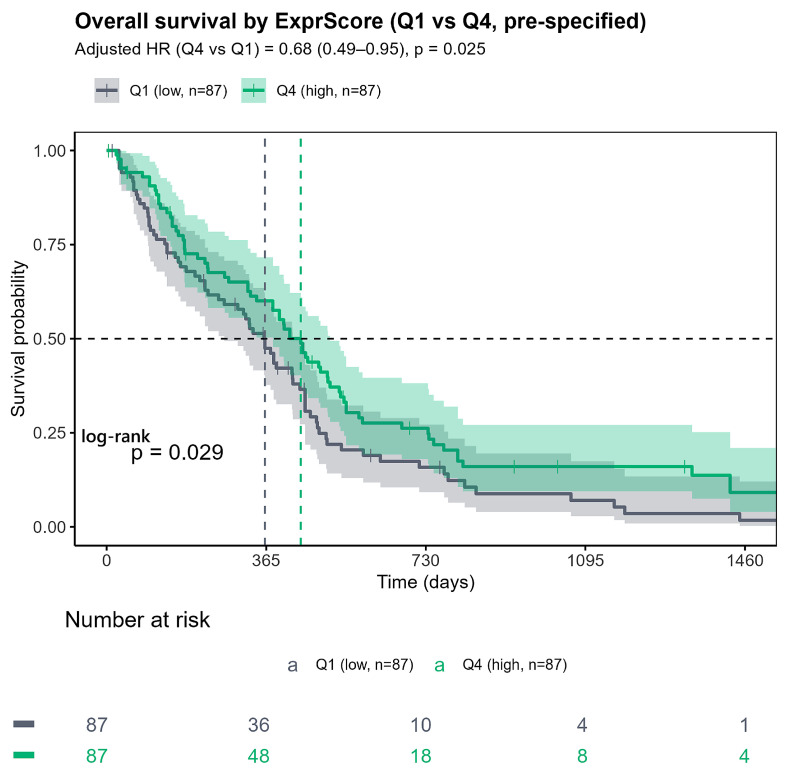
Overall survival by Expression Score (Q4 vs. Q1, pre-specified). Kaplan–Meier curves for high ExprScore (Q4, *n* = 87; green) versus low ExprScore (Q1, *n* = 87; gray). Shaded bands indicate the 95% confidence intervals around the estimated survival at each time point. The numbers at risk below the plot show how many patients remain under follow-up and event-free at the corresponding times. Log-rank *p* = 0.029. In a Cox model adjusted for age and sex, HR = 0.68 (95% CI 0.49–0.95; *p* = 0.025).

To evaluate clinical relevance, we tested survival associations at the set and gene levels. The expression score [ExprScore = mean(Z_HYPO_) − mean(Z_HYPER_)] was prognostic in Cox proportional-hazards models adjusted for age and sex (HR per SD = 0.865; 95% CI 0.769–0.973; *p* = 0.016). A pre-specified quartile comparison (Q4 vs. Q1) confirmed the effect (log-rank *p* = 0.029; adjusted HR = 0.68, 95% CI 0.49–0.95, *p* = 0.025). The Kaplan–Meier display is shown in Figure 7; adjusted survival curves and model outputs are provided in Table S17A–C. On the subset with methylation and overall survival, a higher complementary methylation score (MethScore = β_HYPER_ − β_HYPO_) was associated with poorer OS (adjusted HR per 1-SD increase = 1.73; 95% CI 1.27–2.34; *p* < 0.001), with full results in Table S18A,B.

**Figure 8:**
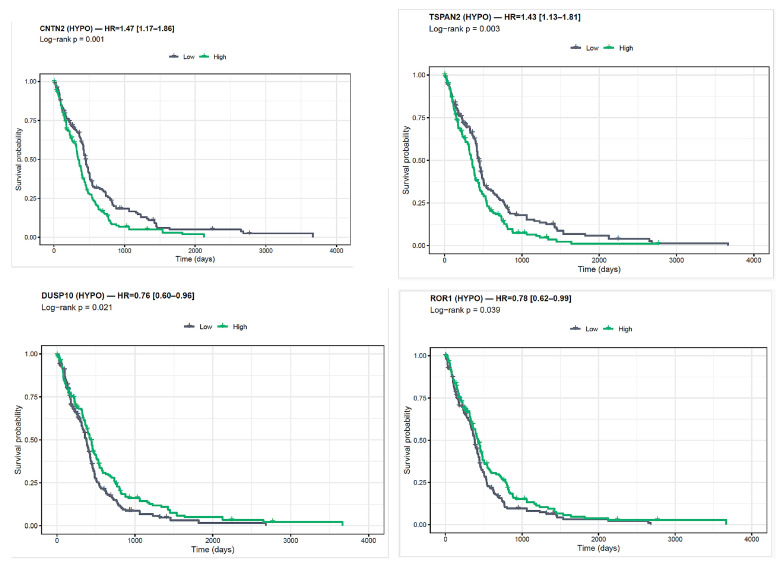
Gene-wise overall survival by expression (TCGA GBM, *IDH*-WT). Kaplan–Meier curves for four promoter-hypomethylated genes. Patients are split at the median expression (High > median, green; Low ≤ median, gray). Lines show KM estimates with 95% CI bands. Panel titles report the Cox HR (High vs. Low, 95% CI), while the *p*-values shown on the plots are from the log-rank test. Higher expression is associated with worse OS for *CNTN2* and *TSPAN2*, and with better OS for *DUSP10* and *ROR1*.

Four promoter-hypomethylated genes showed significant log-rank associations with OS (Figure 8; Table 1): *CNTN2* (HR = 1.47, 95% CI 1.17–1.86; log-rank *p* = 0.001) and *TSPAN2* (HR = 1.43, 1.13–1.81; *p* = 0.003) where higher expression associated with worse outcome; *DUSP10* (HR = 0.76, 0.60–0.96; *p* = 0.021) and *ROR1* (HR = 0.78, 0.62–0.99; *p* = 0.039) where higher expression associated with better outcome. After BH–FDR correction across the 34 genes, *CNTN2* (FDR = 0.038) and *TSPAN2* (FDR = 0.045) remained significant, whereas *DUSP10* (FDR = 0.233) and *ROR1* (FDR = 0.334) were nominal only.

**Figure 9:**
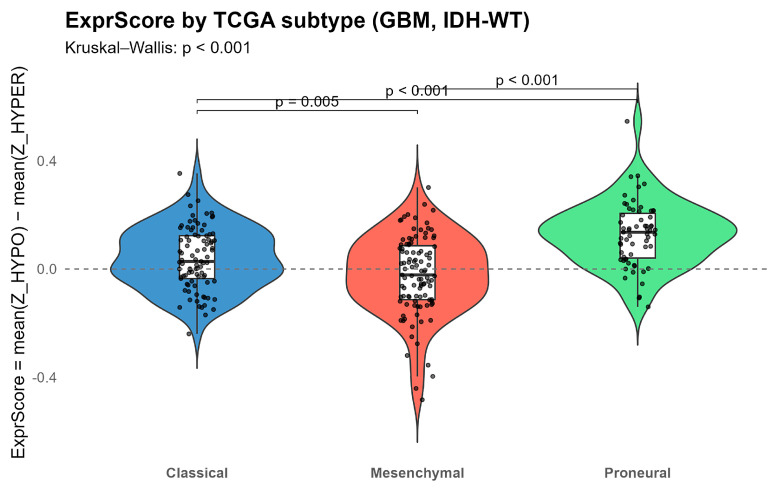
Expression Score across *IDH*-WT glioblastoma subtypes. Violin + box plots show Expressio Score per subtype (Classical, Mesenchymal, Proneural). ExprScore = mean (Z_HYPO) − mean (Z_HYPER); higher values indicate relatively higher expression of HYPO genes and/or lower expression of HYPER genes. Proneural shows the highest ExprScore, whereas Classical and Mesenchymal show lower, comparable ExprScores.

Expression score differed across subtypes (Kruskal–Wallis *p* < 0.001); pairwise was Wilcoxon (BH) showed Proneural > Classical (adjusted *p* = 2 × 10^−5^) and Proneural > Mesenchymal (adjusted *p* = 1 × 10^−9^), while Classical vs. Mesenchymal not significant (Figure 9; Table S19A,B).

Across TCGA GBM *IDH*-wildtype, coding alterations in our 34-gene panel were infrequent: 26/227 (11.45%) tumors harbored ≥1 event, with no gene exceeding 2% (top: *CDKN2C*, *MTOR*, *CNTN2* at 1.8% each), and variants were predominantly missense (Figure S5). These data indicate that recurrent coding mutations in this panel are uncommon, consistent with our focus on epigenetic dysregulation.

## 3. Discussion

This study provides a longitudinal and integrative characterization of promoter DNA methylation within a gliogenesis gene framework in *IDH*-wildtype glioblastoma. Using a curated 48-gene promoter panel derived from a genome-wide gliogenesis ontology, we demonstrate that promoter hypomethylation is the dominant and persistent feature across primary and recurrent tumors. More than half of the analyzed promoters were consistently hypomethylated across all disease stages, while a smaller but stable subset remained hypermethylated. These findings indicate that, despite clinical and therapeutic progression, the gliogenesis-associated promoter landscape in *IDH*-wildtype GBM is conserved mainly, suggesting an epigenetic “core” maintained throughout tumor evolution.

Although the global pattern remained stable, notable heterogeneity emerged at the patient level. Both hypo- and hypermethylation burdens increased significantly with recurrence, and the highest overall burden was observed at second recurrence. The majority of newly gained promoter events occurred during the transition from primary to first recurrence. Beyond this stage, fewer new events were detected, implying stabilization of the methylation state after the first recurrence. Within the hypermethylated subset, the strongest contributors during the primary-to-first recurrence transition included *DAB1*, *VPS54*, *MYOC*, *NCSTN*, and *MTOR*, with substantially fewer additional events at later recurrence. Conversely, within the hypomethylated subset, *CNTN2*, *TAL1*, *TSPAN2*, and *NEGR1* accounted for the majority of newly gained events, again predominantly during the initial recurrence. These results suggest that GBM evolves through early, patient-specific methylation changes that build upon a stable developmental foundation. A plausible explanation is that initial therapeutic interventions, such as radiotherapy and temozolomide-based chemotherapy, together with changes in the tumor microenvironment, impose selective pressures that favor epigenetically reprogrammed subclones. Such therapy-associated epigenetic adaptation has been proposed as a mechanism underlying early clonal selection and treatment resistance in glioblastoma [17]. However, in the present dataset, treatment information is available only at the cohort level (primary tumors sampled prior to therapy and recurrent tumors typically following radiotherapy and/or chemotherapy), which precludes formal correlation analyses with specific treatment regimens or timing. Future longitudinal studies with detailed treatment annotations will be required to directly test this hypothesis.

Klughammer et al. (2018) profiled matched primary and recurrent *IDH*-wildtype glioblastomas and revealed extensive spatial and temporal heterogeneity in DNA methylation, linked to microenvironmental features and patient outcome [18]. These findings establish that the epigenetic landscape of GBM is dynamic and context-dependent, providing a foundation for investigating promoter-level evolution.

Lucas et al. (2025) further showed that *IDH*-wildtype glioblastomas display heterogeneous methylation trajectories across recurrences, with global shifts lacking prognostic value, whereas specific CpG-level changes correlate with survival [19]. This supports the relevance of pathway-focused promoter analyses, such as our gliogenesis-centered approach.

Functional enrichment analyses confirmed the biological relevance of these promoter-level changes. The significant promoter sets were strongly enriched for gliogenesis, glial cell differentiation, neurogenesis, and myelination processes. Both hypo- and hypermethylated subsets captured aspects of glial developmental biology, and Notch-related signaling was among the top pathways in the hypermethylated set. These findings suggest that glioblastoma cells retain and possibly exploit glial lineage programs to maintain their stem-like and proliferative potential. The enrichment of the gliogenic and Notch pathways indicates ongoing epigenetic regulation of developmental genes that may contribute to tumor resistance to treatment.

Drexler et al. (2024) identified a neural-lineage epigenetic signature in high-grade glioma in which hypomethylation of neuronal genes correlated with poorer survival in *IDH*-wildtype glioblastoma, indicating the prognostic relevance of lineage-associated methylation programs [20]. In contrast, Spitzer et al. (2025) reported that recurrent *IDH*-wildtype glioblastomas often shift toward glial- and neural-like cellular states, a pattern associated with prolonged survival [21]. Together, these findings suggest that lineage-linked epigenetic and transcriptional programs remain central to tumor evolution, but their prognostic direction may depend on the specific molecular and cellular context.

In the TCGA validation cohort of *IDH*-wildtype GBM, the promoter-defined categories translated coherently at the transcriptional level. Hypomethylated promoters were associated with higher expression, while hypermethylated promoters were associated with lower expression, consistent with the canonical repressive role of promoter CpG methylation. Significant inverse β–expression coupling (ρ < 0, FDR < 0.05) was observed for 11 genes, including *SOX11*, *TSPAN2*, *CDKN2C*, *TGFB2*, *HES5*, *KCNJ10*, and *HDAC1*, among others. Despite a smaller sample size and incomplete promoter coverage in the 450k + RNA subset, the significant associations maintained the expected direction, confirming that the methylation states identified in the discovery cohort reflect functional transcriptional regulation.

The inverse relationship between promoter CpG methylation and gene expression is well known [22,23]. Zappe et al. (2023) demonstrated that promoter methylation is a central regulatory mechanism, as exemplified by *MGMT*, in which hypermethylation represses transcription and predicts therapeutic response [24]. Extending beyond single genes, Etcheverry et al. (2010) demonstrated genome-wide inverse correlations between promoter methylation and mRNA expression across multiple glioblastoma cohorts, confirming that promoter hypermethylation generally exerts a transcriptional silencing effect [25]. These observations reinforce our finding that hypomethylated promoters in gliogenesis genes are associated with increased expression, thereby validating our interpretation of the methylation–expression coupling results.

Integration of methylation and expression data identified a clinically meaningful epigenetic axis. An expression score contrasting hypomethylated and hypermethylated gene sets [ExprScore = mean(Z_HYPO_) − mean(Z_HYPER_)] was significantly associated with improved overall survival (HR = 0.87; *p* = 0.016), and this association remained robust in quartile-based comparisons (Q4 vs. Q1 HR = 0.68; *p* = 0.025). In the methylation subset, a complementary methylation score (MethScore = β_HYPER_ − β_HYPO_) was associated with a worse prognosis (HR = 1.73; *p* < 0.001), indicating that higher promoter methylation in gliogenesis genes is associated with poorer prognosis. These reciprocal associations between methylation and expression further support the biological coherence of this axis: promoter hypomethylation permits gene activation within the gliogenesis program, whereas hypermethylation represses it, with direct clinical implications.

At the individual gene level, four hypomethylated genes were significantly associated with survival. High expression of *CNTN2* and *TSPAN2* correlated with shorter overall survival, whereas *DUSP10* and *ROR1* expression were associated with more prolonged survival. After correction for multiple testing, *CNTN2* and *TSPAN2* remained significant (FDR < 0.05). Notably, both *CNTN2* and *TSPAN2* were also among the genes most frequently affected by promoter hypomethylation during the primary-to–first recurrence transition in the methylation array dataset, suggesting that their adverse prognostic impact may originate from early epigenetic activation events. While this temporal pattern is compatible with therapy-associated selective pressures, the available data do not allow attribution of these changes to specific treatments. These genes may therefore represent distinct epigenetic nodes within the gliogenic program that carry prognostic and possibly functional relevance. *CNTN2* and *TSPAN2* are involved in neuronal adhesion and axon-glia interactions, processes that may contribute to the invasive and treatment-resistant phenotype of recurrent GBM [26,27].

Beyond their association with survival, experimental and transcriptomic studies provide evidence that *CNTN2* and *TSPAN2* may play active roles in glioma biology. *CNTN2* has been shown to promote glioma stem-like cell proliferation through regulation of EGFR-, HES1-, and APP/AICD-associated signaling pathways, and its knockdown suppresses tumor cell growth in vitro, supporting a functional role in glioblastoma progression [28,29]. In addition, *CNTN2* has been reported to be co-amplified and overexpressed in subsets of malignant gliomas, suggesting that it may confer a selective growth advantage in specific molecular contexts [30].

*TSPAN2*, a tetraspanin enriched in glial lineage cells, has been implicated in oligodendrocyte differentiation, cell adhesion, migration, and neuroinflammatory signaling [27,31]. Altered expression of *TSPAN2* has been reported across glioma transcriptomic and proteomic studies, and modulation of *TSPAN2* influences microglial activation and cytokine signaling, processes increasingly recognized as contributors to the glioblastoma microenvironment and treatment resistance [32]. Together, these findings support the interpretation of *CNTN2* and *TSPAN2* as lineage-associated epigenetic regulators with functional relevance, even in the absence of recurrent coding mutations.

Conceptually, these analyses serve complementary purposes. The expression score is designed as a pathway-level metric to capture coordinated transcriptional activity across gliogenesis genes and to assess its prognostic relevance at the program level. In contrast, single-gene survival analyses were performed to identify individual genes within this program that may act as biologically informative nodes, rather than to establish standalone prognostic markers.

Subtype analysis further investigated the developmental nature of this axis. The expression score was highest in Proneural, intermediate in Classical, and lowest in Mesenchymal tumors (Kruskal–Wallis *p* < 0.001; Proneural > Classical, Proneural > Mesenchymal). This gradient indicates that the expression score reflects a proneural-like transcriptional state, consistent with known biological features of this subtype, including enhanced expression of neural lineage genes and improved overall prognosis [33,34]. The parallel behavior of expression and methylation scores—protective transcriptional activation mirrored by adverse promoter hypermethylation—supports a model in which promoter balance within gliogenesis genes tracks both molecular subtype and clinical outcome. Importantly, the expression score is not intended to replace TCGA subtype classification, but to capture coordinated, pathway-level transcriptional variation within and across subtypes. Its prognostic association, despite partial alignment with subtype structure, suggests that this promoter-linked axis reflects biologically meaningful heterogeneity beyond global subtype assignment.

In our dataset, subtype differences were assessed only in primary *IDH*-wildtype glioblastomas, as recurrent tumors were not available in TCGA. In contrast, Drexler et al. (2024) [13] analyzed matched primary–recurrent pairs of *IDH*-wildtype glioblastomas. They found that about one-quarter of tumors switched DNA methylation subclass at recurrence, most frequently toward a mesenchymal-like profile, without a significant impact on survival [13]. Together, these observations indicate that while global subtype transitions can occur during recurrence, they do not necessarily confer a prognostic advantage, showing that promoter-level analyses can uncover stable, relevant epigenetic patterns.

In another study, only specific DNA methylation subclasses of *IDH*-wildtype glioblastoma, particularly the RTK I/II groups, were shown to benefit from gross total resection, indicating that epigenetic context influences treatment response [35]. This further supports the potential clinical utility of promoter-level axes, such as our gliogenesis program, to complement current subclass-based stratification.

Finally, analysis of coding variants across the same gene panel revealed low mutation frequencies (<2% per gene), confirming that recurrent genetic alterations do not account for the observed effects. The results, therefore, point to epigenetic dysregulation, rather than coding mutation, as the principal correlate of transcriptional and clinical heterogeneity within the gliogenesis program in *IDH*-wildtype GBM.

This study has limitations inherent to retrospective, array-based analyses. Promoter coverage was restricted to annotated CpG probes represented on the Illumina EPIC (850k) and 450k platforms, and recurrent tumors were underrepresented, reducing power to detect subtle longitudinal effects. In addition, detailed treatment annotations (e.g., radiotherapy dose, temozolomide exposure, and timing relative to sampling) were not available at the individual-patient level in the publicly released metadata, precluding formal correlation analyses between specific therapeutic regimens and methylation changes. Methylation–expression coupling was assessed only in primary *IDH*-wildtype glioblastomas, as recurrence datasets with matched transcriptomes are currently limited. Future studies combining single-cell or long-read methylome sequencing with analyses of gliogenesis gene dysregulation will be essential to clarify causal relationships and determine whether promoter-level signatures can guide precision risk assessment and therapy in glioblastoma.

## 4. Materials and Methods

### 4.1. Gene Selection

Genes annotated to the Gene Ontology term GO:0042063 (gliogenesis) were retrieved from AmiGO 2 (v2.5.17) [36] using UniProtKB (https://www.uniprot.org, accessed on 9 August 2025) [37] and a taxon filter for Homo sapiens (NCBITaxon:9606), yielding 333 genes. Gene symbols were standardized to HGNC-approved symbols using HGNChelper in R v4.5.1 [38,39].

### 4.2. Dataset Description

Genome-wide DNA methylation data were obtained from the publicly available GSE279073 dataset in the Gene Expression Omnibus (GEO) repository [40], entitled “Longitudinal epigenome analysis of *IDH*-wildtype glioblastomas from initial and recurrent surgical specimens”. DNA methylation profiling in this cohort was performed on bisulfite-converted genomic DNA using Illumina Infinium HumanMethylationEPIC 850k v1.0 BeadChips (Illumina Inc., San Diego, CA, USA) [19]. The series comprises 226 tumor specimens from 106 patients—99 treatment-naïve primaries and 127 post-therapy recurrences (106 first, 19 s, two third). Third recurrences (*n* = 2) were excluded from inferential analyses a priori and used descriptively in our study.

### 4.3. Methylation Analysis

EPIC array probes from GSE279073 were mapped to the curated gliogenesis universe (GO:0042063; 333 genes). Of these, 328/333 genes were represented by at least one EPIC probe (promoter or non-promoter). DNA methylation levels were quantified using β values, calculated as β = M/(M + U + α), where M and U represent the methylated and unmethylated signal intensities, respectively, and α is an offset (typically set to 100) introduced to stabilize β values when signal intensities are low [41].

Promoter features were defined a priori as Illumina-annotated TSS1500, TSS200, 5′UTR, or 1stExon. For each gene × sample, promoter methylation was summarized as the median β value (across all promoter-mapped probes). To ensure adequate measurement density, we required ≥2 promoter probes per gene, resulting in an inference-ready 48-gene promoter panel. As a sensitivity analysis, promoter-associated probes were additionally analyzed separately by genomic annotation (TSS1500, TSS200, 5′UTR, and first exon), using the same gene-level summarization and statistical criteria and concordance between subregion-specific and promoter-level calls was assessed.

Within each stage, for each gene, we computed the median β value relative to 0.50 using a one-sample Wilcoxon signed-rank test with Benjamini–Hochberg FDR across genes, imposing an effect-size threshold |Δβ| = |β_median_ − 0.50| ≥ 0.20. Calls were: HYPO (hypomethylated; β_median_ ≤ 0.30 and FDR < 0.05), HYPER (hypermethylated; β_median_ ≥ 0.70 and FDR < 0.05), else NS (not significant). Intersections across stages were compiled as presence matrices.

For each patient × stage, we assigned HYPO/HYPER/NS using the stage-wise rules, counted HYPO and HYPER calls per patient, and clustered patients into Low/Mid/High burden groups using k-means (k = 3). Group differences were assessed using a Kruskal–Wallis test with BH-adjusted pairwise Wilcoxon tests. To support the choice of k = 3, we computed average silhouette widths for k = 2–5 separately within each stage, using the two-dimensional burden space (number of hypomethylated and hypermethylated promoters). The value k = 3 showed the highest or near-highest silhouette scores across stages and was therefore retained for consistency across longitudinal analyses.

Longitudinal change on the β scale was computed for P → R1 (primary to first recurrence), R1 → R2 (first to second recurrence), and R2 → R3 (second to third recurrence) only descriptively, as Δβ. We classified HypoGain (Δβ ≤ −0.20), HyperGain (Δβ ≥ +0.20), or Stable, and summed events per gene across transitions. Two-sided binomial tests compared HyperGain with HypoGain at the gene level (BH-FDR across genes).

To model cohort trends while accounting for repeated measures, β was transformed to M-values (M = log2[(β + ε)/(1 − β + ε)], ε = 1 × 10^−6^). We fit linear mixed-effects models (stage as ordinal fixed effect: Primary < First Recurrence < Second Recurrence; patient random intercept) with BH adjustment across genes [42]. As a confirmation on the M-scale, we used limma [43] with duplicateCorrelation (blocking by patient) for First Recurrence vs. Primary and Second Recurrence vs. First Recurrence, with and without TREAT [16] to approximate |Δβ| ≥ 0.20. Cohen’s κ quantified agreement between β-based calls and limma decisions. Leave-one-patient-out re-calling assessed stability (proportion of resamples retaining each call).

We performed over-representation analysis (ORA) with g:Profiler (gprofiler2) [44] on the union of stage-significant promoters and direction-specific subsets (HYPO-only, HYPER-only), using a genome-wide background (GO:BP, Reactome, WikiPathways) [45,46]. BH-FDR ≤ 0.05 defined significance. As part of the sensitivity analyses, ORA was repeated with restricted backgrounds (the 48-gene panel and the 333-gene gliogenesis universe).

### 4.4. TCGA Analyses

TCGA-GBM bulk RNA-seq counts, clinical data (overall survival; progression-free interval when available), somatic variants (MAF), and DNA methylation data generated using the Illumina HumanMethylation450 BeadChip (Illumina Inc., San Diego, CA, USA) were obtained from the NCI’s Genomic Data Commons (GDC). Analyses were restricted to primary tumors and *IDH*-WT cases (pathogenic *IDH1*/*IDH2* mutations excluded by MAF), yielding *n* = 347 with RNA-seq and overall survival. RNA-seq counts were normalized by edgeR/TMM [47] and transformed to logCPM after low-expression filtering. A matched subset had RNA-seq + 450k (*n* = 74) for methylation–expression coupling; a separate subset had 450k + OS (*n* = 97) for methylation-based survival.

#### 4.4.1. Set-Level Expression Coherence

From the promoter analysis, we defined HYPO and HYPER gene sets. Across primary and recurrent stages, a total of 34 unique genes were classified as significantly hypomethylated or hypermethylated in at least one stage and had available RNA-seq expression data in TCGA *IDH*-wildtype glioblastoma; this union set was used for expression-based analyses. For each sample, we computed in-sample Z-scores for these genes and an expression score, defined as ExprScore = mean(Z_HYPO_) − mean(Z_HYPER_). Cohort coherence was assessed using a paired Wilcoxon test (within-sample Z_HYPO_ vs. Z_HYPER_). At the gene level, one-sample Wilcoxon tests on Z (expected: HYPO > 0, HYPER < 0) were adjusted for multiple comparisons using the BH-FDR method.

#### 4.4.2. Methylation–Expression Coupling (*n* = 74)

Promoter methylation per gene was summarized as median β value (median of TSS1500/TSS200/5′UTR/1stExon probes). For each gene, we computed Spearman’s ρ between median β and expression and adjusted *p*-values by BH-FDR. Significant coupling was defined as FDR < 0.05 with an effect-size filter |ρ| ≥ 0.30; the a priori expectation was ρ < 0 (repressive promoter mark).

#### 4.4.3. Survival Modeling

The primary endpoint was overall survival (OS). At the set level, Cox models adjusted for age and sex used expression score as a standardized continuous covariate (per 1 standard deviation—SD), and a pre-specified comparison between the highest and lowest quartiles (Q4 vs. Q1) contrast was evaluated with KM + log-rank (*p*-value) and adjusted Cox (hazard ratio—HR for Q4 vs. Q1). In the methylation−OS subset (n ≈ 97), we defined the methylation score (MethScore) as mean(β_HYPER_) − mean(β_HYPO_) and fit adjusted Cox models per 1 SD.

For per-gene survival, expression was dichotomized at the cohort median (High > median vs. Low ≤ median). We used KM + log-rank for the *p*-value and a binary Cox model to report the HR (High vs. Low) with 95% CI. Multiple testing across the 34 gene-wise median-split tests was controlled by Benjamini–Hochberg FDR applied to the log-rank *p*-values. Tests were two-sided.

Transcriptional subtype labels (classical, mesenchymal, proneural) were taken as provided by TCGA/Verhaak (no re-classification). Associations between subtype and expression score (and HYPO/HYPER ssGSEA scores) were tested using Kruskal–Wallis, followed by BH-adjusted pairwise Wilcoxon tests.

#### 4.4.4. Somatic Mutations (Exploratory)

Non-synonymous variants in the 34-gene panel (missense, nonsense, frameshift, and splice-site) were summarized with maftools (oncoplots, frequencies) using tumors with available MAFs (*n* = 227), without filtering by predicted pathogenicity, as the analysis was intended to be descriptive.

All analyses were conducted in R (version 4.5.1) using RStudio. For the GSE methylation workflow, we used base R statistical functions (Wilcoxon, Kruskal–Wallis) and standard CRAN/Bioconductor packages, including lme4/lmerTest (mixed-effects models), limma (duplicateCorrelation, TREAT), and gprofiler2 (over-representation analysis). For the TCGA workflow, we used edgeR (TMM normalization, logCPM), survival (Cox models, Kaplan–Meier analysis, Schoenfeld diagnostics), GSVA (ssGSEA), and maftools. Benjamini–Hochberg FDR was used throughout for multiple-testing control.

## 5. Conclusions

This study identifies a stable yet clinically informative epigenetic signature centered on gliogenesis promoters. The methylation landscape remains largely conserved across disease progression, but the relative balance of hypo- and hypermethylation across this network captures meaningful transcriptional and prognostic variation. Together, these findings delineate a coherent epigenetic axis that links developmental lineage regulation to tumor behavior and patient survival. Promoter hypomethylation of gliogenesis genes is transcriptionally activating and prognostically favorable, whereas progressive hypermethylation is associated with a poorer outcome. This framework shows the potential of promoter-level methylation profiling to complement transcriptomic classifiers and refine risk stratification in *IDH*-wildtype glioblastoma.

## Figures and Tables

**Table 1:** Survival associations (log-rank significant, *p* < 0.05).

Gene	Set	HR (95% CI)	*p*-Value (Log-Rank)	FDR (BH)	Prognosis (High Expr)
*CNTN2*	HYPO	1.47 (1.17–1.86)	0.001	0.038	High worse
*TSPAN2*	HYPO	1.43 (1.13–1.81)	0.003	0.045	High worse
*DUSP10*	HYPO	0.76 (0.60–0.96)	0.021	0.233	High better
*ROR1*	HYPO	0.78 (0.62–0.99)	0.039	0.334	High better

Notes: HR is High vs. Low expression group with 95% CI; *p*-values are log-rank tests with BH-adjusted FDR.

## Data Availability

The original contributions presented in this study are included in the article/Supplementary Material. Further inquiries can be directed to the corresponding author.

## Supplementary Materials

The following supporting information can be downloaded at: https://www.mdpi.com/article/10.3390/ijms27021112/s1.
