# Supplementary figures and images for: Promoter Methylation–Expression Coupling of Gliogenesis Genes in IDH-Wildtype Glioblastoma: Longitudinal Analysis and Prognostic Value
Source: Int J Mol Sci. 2026 Jan 22;27(2):1112. doi: 10.3390/ijms27021112 (PMC12841690; doi:10.3390/ijms27021112)

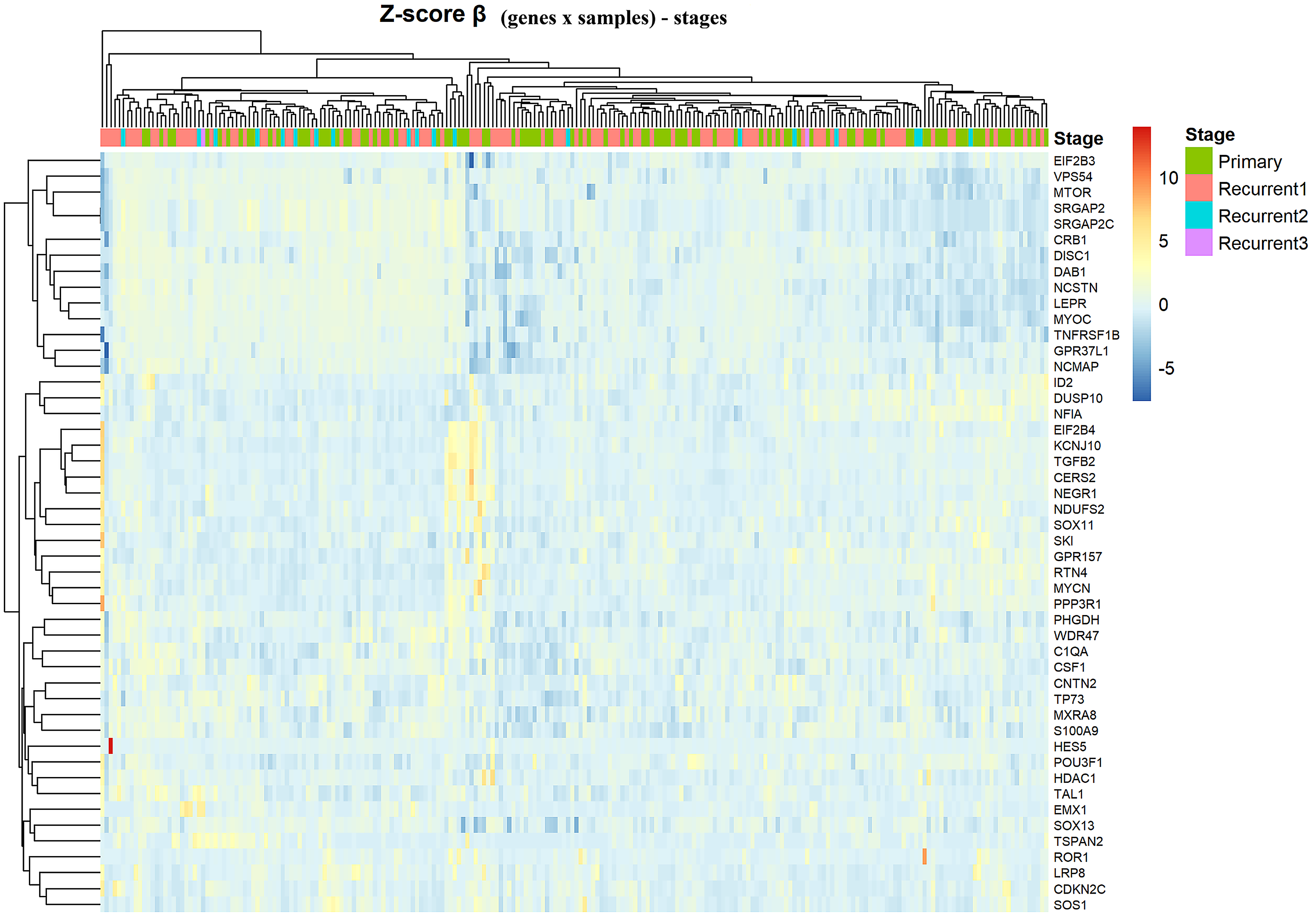

Supplement: Supplementary file 1 [file ijms-27-01112-s001.zip › FigureS1. Z-scored promoter β across stages.png]

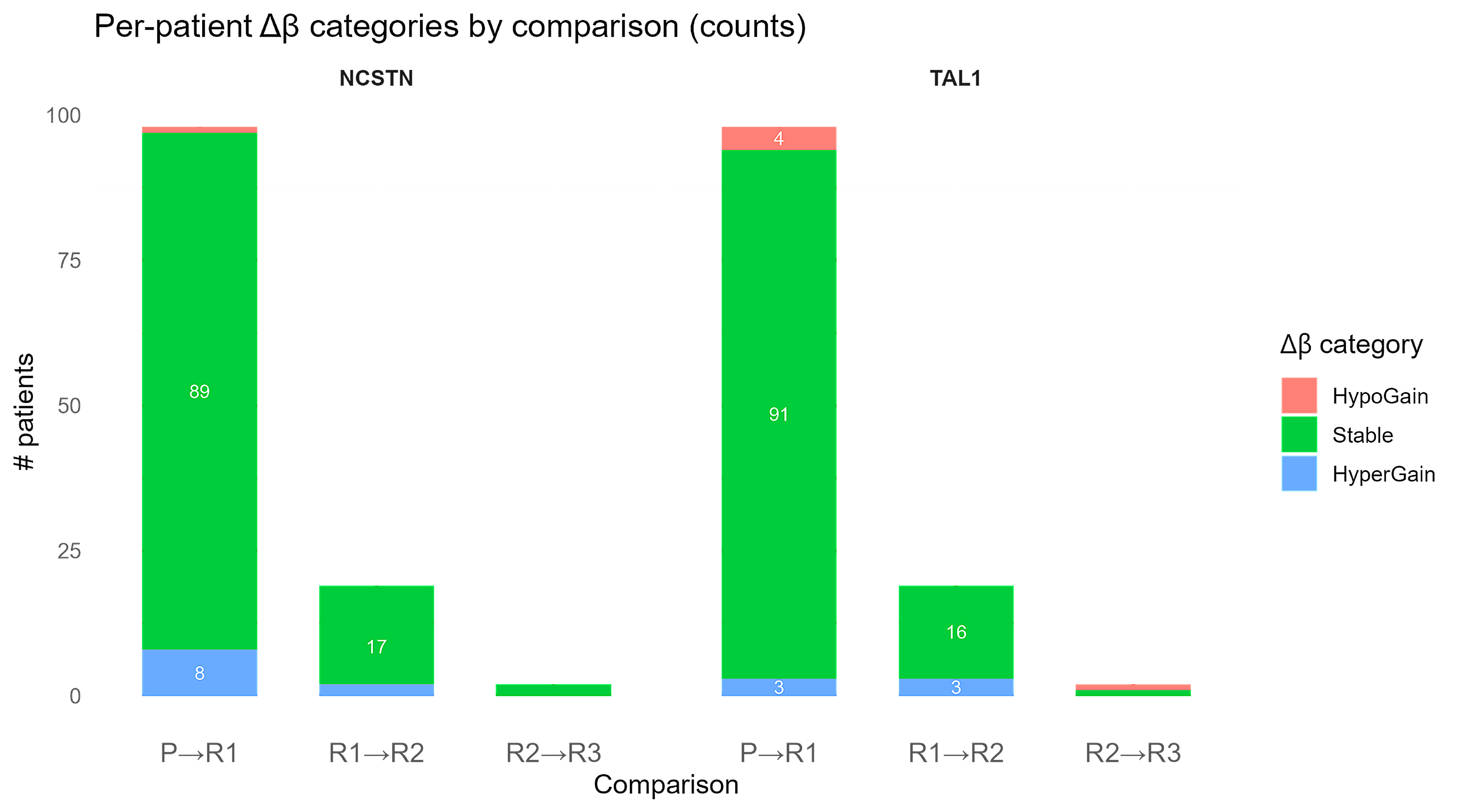

Supplement: Supplementary file 1 [file ijms-27-01112-s001.zip › FigureS2. Paired patient-level changes for NCSTN and TAL1.png]

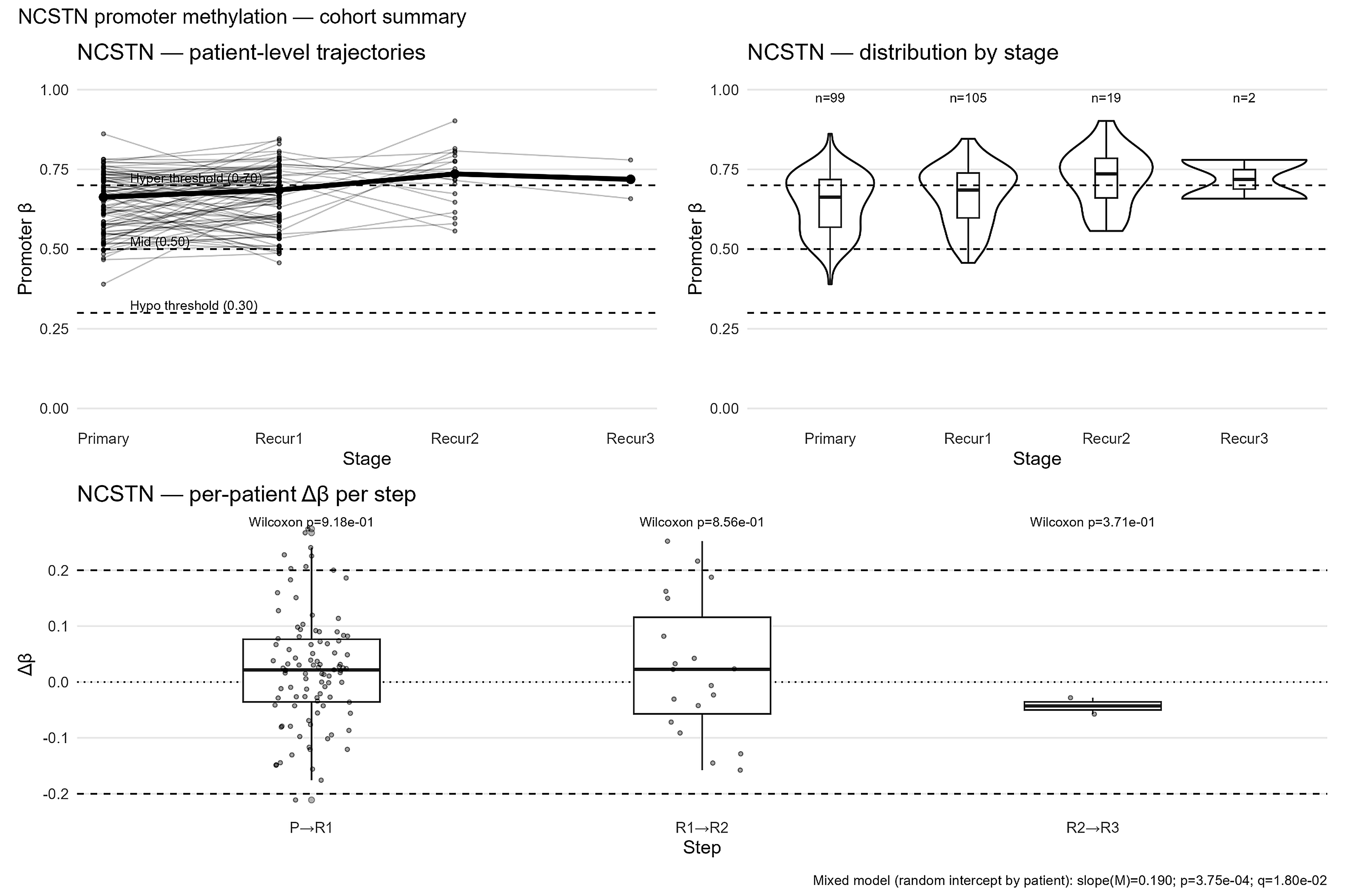

Supplement: Supplementary file 1 [file ijms-27-01112-s001.zip › FigureS3. NCSTN promoter β over time (cohort summary).png]

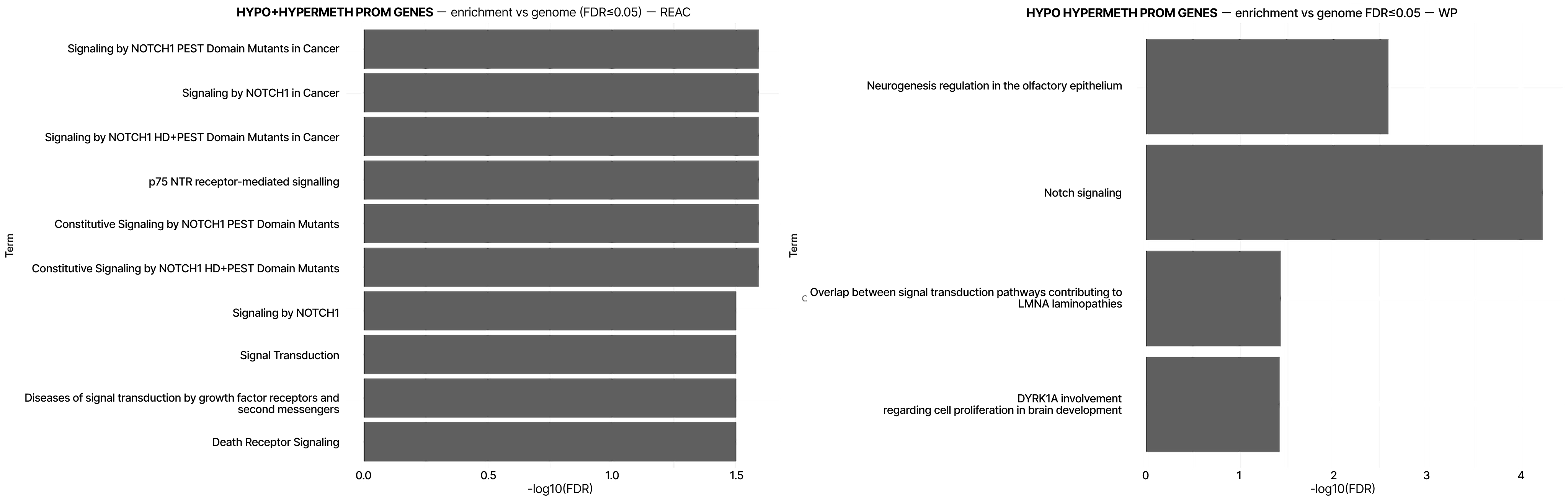

Supplement: Supplementary file 1 [file ijms-27-01112-s001.zip › FigureS4. Pathway enrichment for significant promoter-methylated genes.png]

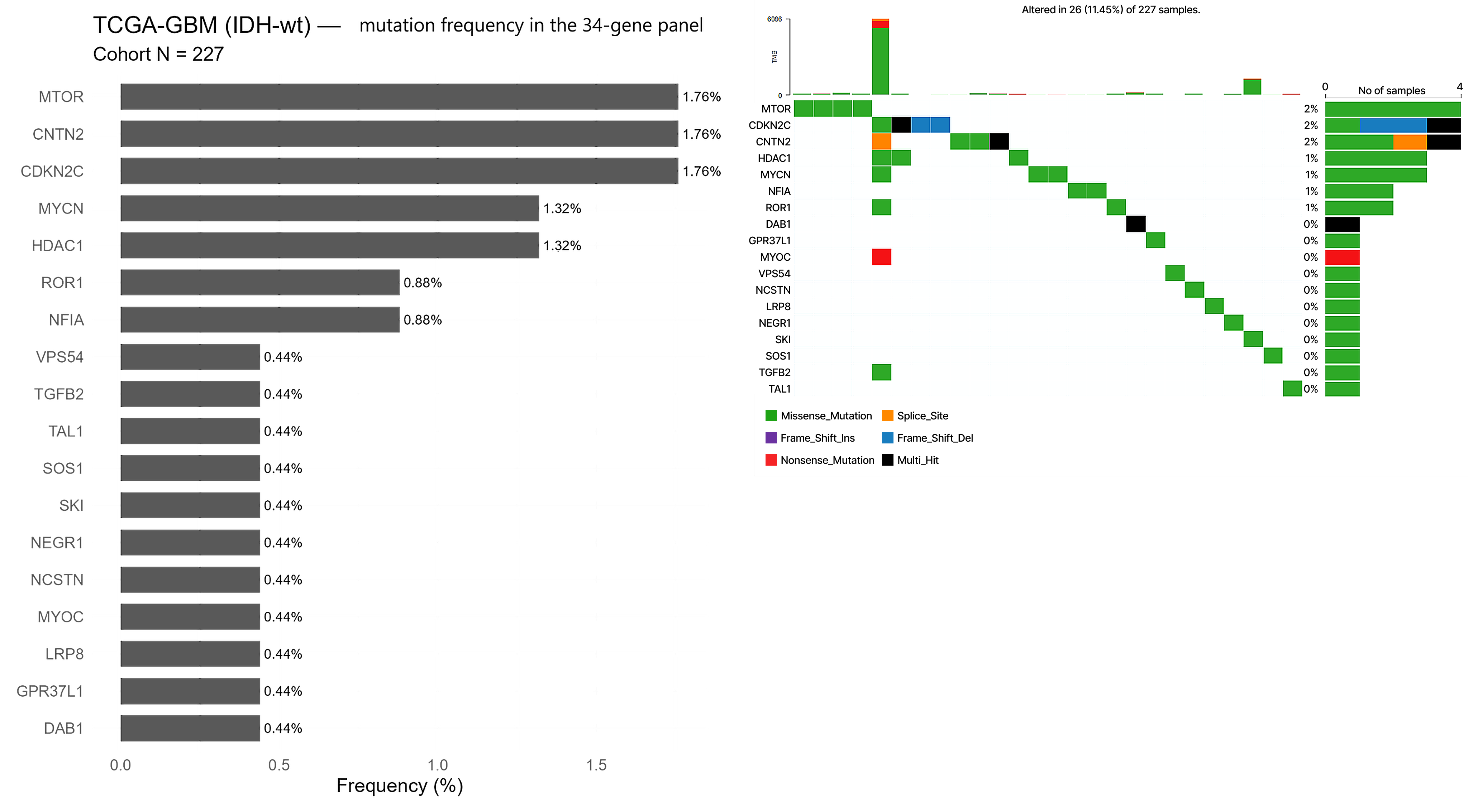

Supplement: Supplementary file 1 [file ijms-27-01112-s001.zip › FigureS5. Mutation landscape of the 34-gene panel (TCGA GBM, IDH-WT).png]
